# New Insights on the Toxicity on Heart and Vessels of Breast Cancer Therapies

**DOI:** 10.3390/medsci10020027

**Published:** 2022-05-25

**Authors:** Oreste Lanza, Armando Ferrera, Simone Reale, Giorgio Solfanelli, Mattia Petrungaro, Giacomo Tini Melato, Massimo Volpe, Allegra Battistoni

**Affiliations:** Department of Clinical and Molecular Medicine, Sapienza University of Rome, 1035-1039 Rome, Italy; oreste.lanza@uniroma1.it (O.L.); armando.ferrera95@gmail.com (A.F.); sreale@ospedalesantandrea.it (S.R.); giorgiosolfanelli@gmail.com (G.S.); mattia.petrungaro@uniroma1.it (M.P.); giacomo.tinimelato@uniroma1.it (G.T.M.); massimo.volpe@uniroma1.it (M.V.)

**Keywords:** breast cancer, cardio-oncology, chemotherapy, cardiotoxicity, chemotherapy-induced cardiotoxicity

## Abstract

Cardiovascular diseases are largely represented in patients with cancer and appear to be important side effects of cancer treatments, heavily affecting quality of life and leading to premature morbidity and death among cancer survivors. In particular, treatments for breast cancer have been shown to potentially play serious detrimental effects on cardiovascular health. This review aims to explore the available literature on breast cancer therapy-induced side effects on heart and vessels, illustrating the molecular mechanisms of cardiotoxicity known so far. Moreover, principles of cardiovascular risk assessment and management of cardiotoxicity in clinical practice will also be elucidated. Chemotherapy (anthracycline, taxanes, cyclophosphamide and 5-fluorouracil), hormonal therapy (estrogen receptor modulator and gonadotropin or luteinizing releasing hormone agonists) and targeted therapy (epidermal growth factor receptor 2 and Cyclin-dependent kinases 4 and 6 inhibitors) adverse events include arterial and pulmonary hypertension, supraventricular and ventricular arrhythmias, systolic and diastolic cardiac dysfunction and coronary artery diseases due to different and still not well-dissected molecular pathways. Therefore, cardiovascular prevention programs and treatment of cardiotoxicity appear to be crucial to improve morbidity and mortality of cancer survivors.

## 1. Background

Breast cancer is the most common cancer among women and one of the most significant causes of death, representing a major public health problem [1,2]. Between 2015 and 2020, 7.8 million women were diagnosed with breast cancer, this cancer being the most common in the world [3]. It affects women at any age after puberty in every country of the world, with increasing rates in later life. Due to early detection programs, as well as advances in treatment, death rates for breast cancer have recently been declining, but this cancer still represents a leading cause of deaths in 119 countries [4]. Survivor women present more lost disability-adjusted life years (DALYs) than any other type of cancer globally, which is also due to increased morbidity and to adverse effects of chemotherapies [3]. Cardiovascular diseases (CVD) are one of the most important side effects of systemic breast cancer treatment that may heavily affect quality of life and lead to premature morbidity and death among breast cancer survivors [5]. Systemic therapy is defined as the use of medication to destroy cancer cells and includes chemotherapy, hormonal therapy and targeted therapy.

### 1.1. Chemotherapy

Chemotherapy regimen is defined as a combination of drugs given in a specific number of cycles over a set period. Many types of chemotherapy, with different mechanisms of action, have proven to be effective in treating breast cancer, depending on its grading, staging and patient comorbidities. Commonly used regimens include anthracyclines, such as doxorubicin, epirubicin and pegylated liposomal doxorubicin; taxanes, such as docetaxel and paclitaxel; cyclophosphamide; and 5-fluorouracil (5-FU) [6].

### 1.2. Hormonal Therapy

Hormonal therapy is an effective treatment for neoplasms expressing estrogen (ER positive) and/or progesterone (PR positive) receptors (79% of all breast cancer [7]), which use hormones to fuel their growth. Blocking receptors can help prevent cancer recurrence and death. Hormonal therapy might be used either alone or after chemotherapy. Hormonal therapy includes estrogen receptor modulators, such as tamoxifen, aromatase inhibitors (AI) and gonadotropin, or luteinizing releasing hormone (GnRH or LHRH) agonists, such as goserelin and leuprolide [8].

### 1.3. Targeted Therapy

Targeted therapy is a treatment specifically targeting cancer genes, proteins, or the tissue environment that contributes to cancer growth and survival. In treating breast cancer, one of the most important targets is the HER2 receptor that is overexpressed in 15–18% of all breast cancers. Therapies that target the HER2 receptor may be given along with chemotherapy and include: Trastuzumab, Pertuzumab, Ado-trastuzumab emtansine (T-DM1) and Lapatinib [9]. Other important pharmacological targets are Cyclin-dependent kinases 4 and 6 (CDK4/6). These drugs include abemaciclib, palbociclib and ribociclib [10].

## 2. Methods

We performed a comprehensive literature search for data on the prevalence, pathophysiologic mechanisms, diagnosis, treatment implications and preventive strategies of cardiotoxicity in patients under therapy for breast cancer.

We included the databases on PubMed and MEDLINE, using search terms for a range of conventional, adjunct and novel cancer therapeutics plus radiotherapy. The range of cancer therapies were searched against the terms “cardiovascular disease”, “cardiovascular prevention” “cardiotoxicity”, “cardio-oncology” and “heart failure”. Inclusion criteria were articles published from 2000 to 1st March 2022, in English.

Original articles and meta-analysis have been reviewed, selecting most recent papers and those with the largest sample size.

Reviews and consensus papers were included when deemed relevant and related to the topic. Moreover, we expanded results by a manual search in the references of selected reports to identify additional relevant information.

## 3. Chemotherapy Induced Cardiotoxicity

Cardiac toxicity is a major and worrying side effect of chemotherapies. Indeed, different regimens of treatment might lead to left ventricular (LV) dysfunction and heart failure, ischemic heart disease, hypertension, thromboembolism, pericarditis and myocarditis and arrhythmias [11]. LV dysfunction is of interest due to its high incidence and severe prognosis among patients affected by breast cancer and treated with anthracyclines. In this setting, different definitions of cardiotoxicity have been proposed so far, all including a decline in the left ventricular ejection fraction (LVEF), detected by different methods (Table 1). One of the first definitions was proposed more than forty years ago by Alexander et al. [12], with “mild” cardiotoxicity being a decline, detected by multigated acquisition (MUGA) scanning, in LVEF > 10%, “moderate” being a decline in LVEF > 15% to final LVEF < 45% and “severe” if symptoms of congestive heart failure are present. In 2016, the European Society of Cardiology [13] defined cardiotoxicity as a decline in LVEF of at least 10% to a final value of under 53% in repeated evaluations (echocardiography, cardiac magnetic resonance imaging and MUGA scan). With regards to LV dysfunction, age and pre-existing LV dysfunction are two of the most important risk factors associated with the development of cardiotoxicity, but other CV conditions, including arterial hypertension, diabetes and coronary artery disease, are also associated with the increased risk of cardiotoxicity [14]. Moreover, it has been shown that African Americans are at a higher risk of developing cardiotoxicity than Caucasian [15]. Pharmacogenomics is also emerging as a potential topic to help identify patients who are at higher risk for cardiotoxicity [16]. Furthermore treatment-related risk factors, including a higher dose of chemotherapy or specific formulations, and additional agents or radiation might also increase the risk of cardiotoxicity [14] (Table 2 and Table 3). 

### 3.1. Anthracyclines

Anthracyclines are cytostatic antibiotics extracted from Streptomyces bacterium [21]. An anthracycline-based chemotherapy is used in about one third of patients affected by breast cancer ≥66 years old and in half of patients ≤65 years old [22]. The most used anthracyclines are doxorubicin, daunorubicin, epirubicin and idarubicin. These compounds might also be used to treat other cancers, including leukemias, lymphomas, stomach, uterine, ovarian, bladder and lung cancer. Anthracyclines have many different mechanisms of action on cancer cells, including free radical formation [23], lipid peroxidation, direct membrane effects and enzyme interactions [24]. The most important mechanism seems to be the interaction with topoisomerase II, a complex that promotes chromosome disentanglement. By inhibiting this complex, anthracyclines promote growth arrest and apoptotic cancer cell death [25]. Unfortunately, CV anthracyclines induced cardiotoxicity might also develop. It can be classified as acute, early onset chronic or late onset chronic. Acute cardiotoxicity occurs after a single dose, or a single course, in <1% of patients, with the onset of symptoms within 14 days from the end of treatment and is usually reversible. Usually, it presents with supraventricular arrhythmia, transient LV dysfunction and electrocardiographic (ECG) changes [26]. Early-onset chronic cardiotoxicity is the most common type of cardiotoxicity. It occurs within 1 year of treatment with dilated-hypokinetic cardiomyopathy and the progressive evolution towards heart failure. Late-onset chronic cardiotoxicity develops after years (a median of 7 years after treatment) and its clinical presentation is similar to the early-onset chronic cardiotoxicity. The two chronic forms are considered irreversible with a poor prognosis [27]. The anthracyclines-related cardiotoxicity is dose-dependent. Indeed, doxorubicin is associated with a 5% incidence of congestive heart failure when a cumulative lifetime dose of 400 mg/m^2^ is reached, whereas higher doses lead to an increasing risk [13] (Table 4). Epirubicin and liposomal anthracyclines have been reported to be less cardiotoxic than doxorubicin, which has comparable antitumor activity. Mao et al. show in their meta-analysis that epirubicin was probably more toxic than liposomal doxorubicin with an OR of 1.87 (CI 95% 0.98–3.57) but less toxic than doxorubicin, which was associated with the highest rates of cardiac adverse effect with an OR compared to epirubicin of 1.84 (1.18, 2.93) [28].

**Figure 1:**
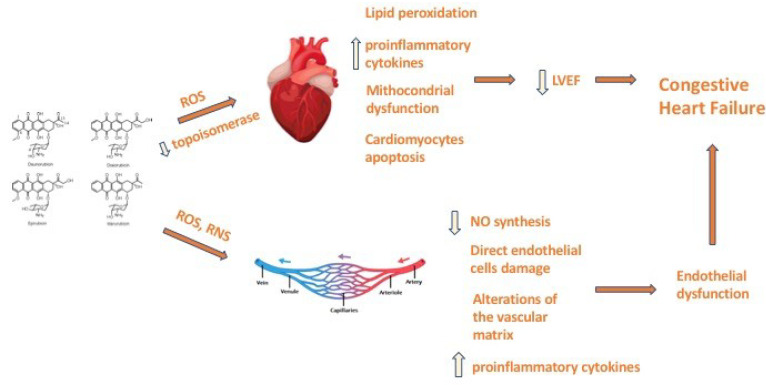
Molecular mechanisms of anthracyclines induced cardiotoxicity. LVEF, left ventricle ejection fraction; NO, nitric oxide; RNS, reactive nitrogen species; ROS, reactive oxygen species.

Liposomal doxorubicin formulations (liposomal doxorubicin and pegylated liposomal doxorubicin) are encapsulated phospholipid membrane drugs. These drugs show comparable efficacy with conventional anthracyclines, but they appear to be safer. Indeed, this kind of formulation determines lower levels of free drugs in the blood and fewer nonspecific bindings when compared to conventional doxorubicin. In fact, the large size of the liposome vesicles reduce doxorubicin exposure to cardiac tissues and mononuclear phagocytes may recognize the larger size of the liposomes more easily, improving the clearance of the drugs [29]. Cardiomyocytes are the main target of anthracycline toxicity, leading to a progressive development of cardiac dysfunction. However, other cell types, such as endothelial cells, cardiac progenitor cells and cardiac fibroblasts, have been identified as potential additional targets, creating a more complex scenario in the pathogenesis of anthracycline-induced cardiotoxicity (Figure 1). Moreover, Novo et al. [30] demonstrate an increase in arterial stiffness in patients treated with anthracyclines. Indeed, anthracycline-induced endothelial vascular damage might provoke structural acute vascular changes through the alterations of the vascular matrix and by interfering with the endothelial regulation of vascular smooth muscle cell tone by reducing nitric oxide synthesis. Furthermore, reactive nitrogen species might act together with reactive oxygen species to directly damage endothelial cells, causing nitrosative stress. Anthracyclines may also promote the overexpression of proinflammatory cytokines that can further cause endothelial damage [31].

Therefore, in patients at low risk for cardiotoxicity guidelines recommend an echocardiographic assessment and measuring cardiac biomarkers (BNP or NT-proBNP and cardiac troponin), at least at baseline and after 12 months from the final cycle of chemotherapy.

In patients at medium risk for cardiotoxicity, guidelines recommend an echocardiographic assessment and the measurement of cardiac biomarkers at least at baseline, before the 5th cycle of chemotherapy (but preferably before every cycle) and 12 months after the last cycle of chemotherapy. In patients at high risk for cardiotoxicity, an echocardiographic assessment and the measurement of cardiac biomarkers is recommended at baseline, before the 2th, 4th and 6th cycle (but preferably before every cycle) and after 3, 6 and 12 months after the last cycle of chemotherapy [32].

In patients at high risk for cardiotoxicity, guidelines recommend the use of liposome-encapsulated doxorubicin and the use of an appropriate cardioprotective regimen as dexrazoxane, beta-blockers (preferably carvedilol), angiotensin-converting enzyme inhibitors (ACEi) (preferably enalapril) and angiotensin II receptor blockers (ARBs) [13] to minimize cardiotoxicity. 

### 3.2. Taxanes

Taxanes (paclitaxel and docetaxel) are chemotherapeutic agents used in about 50% of patients affected by breast cancer [22]. They produce antitumor activity by binding tubulin and stabilizing cellular microtubules, thereby inhibiting cancer cell division. Cardiotoxicity is usually observed when taxanes are used in combination with anthracyclines [26]. This is due to pharmacokinetic interference of anthracycline elimination by the taxanes, which increase the cardiotoxic effects of anthracyclines and promoting higher plasma levels of anthracyclines [33]. In this setting, paclitaxel is more cardiotoxic than docetaxel. Therefore, taxane-induced cardiotoxicity typically presents with congestive heart failure [34]. Taxanes, however, might also promote cardiac dysfunction regardless of anthracycline, increasing oxidative stress and causing increased arterial stiffness inducing senescence in vascular endothelial cells, which coincides with decreased activity of endothelial nitric oxide synthase (eNOS) in these cells [35]. Taxanes might also cause cardiac arrhythmias, such as sinus bradycardia (especially paclitaxel), atrioventricular block and atrial fibrillation. Cardiac arrhythmias induced by taxanes are usually benign and without symptoms. However, cardiac monitoring is usually recommended during the first hours of infusion of taxanes [36].

### 3.3. Cyclophosphamide

Cyclophosphamide (CP), an alkylating nitrogen mustard with strong antineoplastic activity and immunosuppressive activity [37], is to date widely used to treat different types of cancers, including breast cancer at different stages, as adjuvant therapy in the beginning or in metastatic disease, augmenting response rate, time to disease progression and overall survival. 

CP is usually effective, but its wide clinical application is currently limited by its toxicity, generally dose-dependent and reversible with proper medical treatment. Indeed, although CP is, to some extent, well tolerated at lower doses, high-dose regimens, such as those given in breast cancer treatment, can cause a variety of adverse effects. Previous anthracycline treatment or mediastinal radiation therapy, age above 50 years old and the existing presence of LV dysfunction seem to be risk factors. CP-induced cardiotoxicity varies from 7 to 28% and mortality ranges from 11 to 43% at the therapeutic dose of 170–180 mg/kg, i.v, with an onset a few days after treatment. The exact mechanism of CP-induced cardiotoxicity has not been well established [38]. CP undergoes hepatic metabolism by cytochrome P-450 with metabolites causing oxidative stress and direct endothelial capillary harm [39]. The newly formed aldophosphamide decomposes into phosphoramide mustard, an active antineoplastic agent, and acrolein, a toxic metabolite which acts on the myocardium and endothelial cells [38,40]. The endothelial cells are ruptured, leading to interstitial hemorrhage, edema, damage to myocytes and the development of intracapillary microthrombi, resulting in ischemic damage [41]. Common symptoms of CP-induced cardiotoxicity may include heart failure, myocarditis, tachyarrhythmias, hypotension and pericardial disease [42]. However, the classic CP cardiotoxicity is represented by an acute form of myo-pericarditis, usually associated with a higher dose therapy [43]. The cardiotoxicity of CP might also manifest as a reduction in left ventricular systolic function. Once recognized, the treatment of CP induced cardiotoxicity is based on drugs used in the treatment and the prevention of heart failure, such as diuretics, angiotensin-converting enzyme inhibitors and β-blockers, which can also be prescribed earlier if there are no major contraindications. Mild to moderate heart failure and small pericardial effusions may generally resolve within a short while after stopping CP administration, while severe scenarios may end in irreversible heart dysfunction [44].

### 3.4. 5-Fluorouracil

The chemotherapeutic agent 5-fluorouracil (5-FU), a fluoropyrimidine, synthetic antimetabolite, is commonly used in the treatment of a wide variety of solid tumors, including breast cancer. 5-FU is the second most common cause of cardiotoxicity after anthracyclines. Cardiac symptoms generally occur early during the drug infusion. The median onset time is 12 h following infusion, though cardiotoxicity is reported to occur anytime during infusion or even up to 1–2 days after infusion [45]. A meta-analysis revealed an incidence of symptomatic cardiotoxicity of 1.2 to 4.3% during treatment with 5-FU and outlined how the risk can be augmented by continuous infusion and concurrent treatment with alkylating agents, such as cisplatin [46]. Patients with CV comorbidities may be at increased risk. The mechanism of 5-FU-related cardiotoxicity is poorly understood. The two most likely contributors are ischemia and/or coronary vasospasm and direct myocardial toxicity. Myocardial ischemia [47] may vary from angina pectoris to acute myocardial infarction (MI) and can occur in patients with an incidence of 1.1%, and up to 15.1% for patients affected by previous ischemic heart disease. Coronary vasospasm remains the most well-established mechanism of fluoropyrimidine related myocardial ischemia. It can be directly visualized during coronary angiography, associated with brachial artery vasoconstriction immediately following the administration of 5-FU [48]. In case of acute cardiotoxicity induced by 5-FU, chemotherapy suspension is recommended, followed by treatment with aspirin, calcium channel blockers and long-acting nitrates. It should be noted that the evidence of significant coronary stenosis may not exclude the plausibility of overlapping 5-FU-related cardiotoxicity and any re-administration of 5-FU must be adopted cautiously with strict monitoring. Other less common manifestations of cardiotoxicity include supraventricular arrhythmias [49], myocarditis and pericarditis [50] and heart failure [51]. In general, the reintroduction of the 5-FU after an established cardiotoxic event is not considered safe due to the risk of recurrence associated with complications, if alternative chemotherapy regimens of equivalent efficacy are available. 

## 4. Hormonal Therapy

### Estrogen Receptor modulators and Aromatase Inhibitors

Endocrine therapy with selective estrogen receptor modulators (SERM), such as tamoxifen and AI, plays an important role in the treatment of breast cancer overexpressing hormone receptors, such as ER and PR receptors [52]. These drugs can inhibit the hormone signaling, which is responsible for the uncontrolled cell growth in such forms of breast cancer. A reduction in the cancer recurrence rate and increased overall survival has been demonstrated by using endocrine therapy for an extended period, usually five years [53]. 

Tamoxifen is the most widely used SERM. Its effects in different tissues mainly depend on the presence of a coactivator or corepressor that can bind to the complex tamoxifen/ER receptor [54]. In breast tissue, it acts by inhibiting cell growth, but in the endometrium, in bones or other tissues it might work as an ER agonist [54]. In the CV system, due to its agonist function, tamoxifen could have a cardioprotective effect, probably due to the lowering of total cholesterol and low-density lipoprotein (LDL) cholesterol by inhibiting the activity of some enzyme in the cholesterol pathway, such as AcetylCo-A acetyltransferase [55]. In some studies, it has been proposed that tamoxifen can have anti-inflammatory effects, eliciting the activation of the transforming growth factor Beta (TGF-β) pathway [56,57]. Moreover, tamoxifen seems to have an antioxidant effect that can reduce the harmful oxidation of LDL [58]. Despite this positive effect in postmenopausal women with breast cancer, numerous studies failed to demonstrate a relevant effect of tamoxifen in preventing CVD and CV death [59]. Moreover, the agonist activity of tamoxifen might cause enhanced thrombogenicity in postmenopausal women [59]. In the early 1990s, Saphner et al. observed significant major venous thromboembolic complications in patients treated with tamoxifen compared to patients not taking tamoxifen in therapeutic schemes [60]. Amir et al., in a 2011 metanalysis, demonstrated that the incidence of deep vein thrombosis in tamoxifen treated patients was higher than in patients assuming AI [61]. Deep vein thrombosis might complicate with pulmonary embolism, which is a worrisome adverse side effect of tamoxifen. Finally, in 2018, Grouthier et al. revealed an increased incidence of QT prolongation, torsade de pointes and ventricular arrhythmias in patients on SERM (tamoxifen and toremifen) treatment compared to AI [62].

AI are a class of drugs that can reduce the conversion of androstenedione to estradiol, thus reducing the circulating levels of estrogens, decreasing the possibility of tumor cell growth [63]. Anastrazol, letrozol and exemestane can be used as upfront strategy or in a sequential treatment with tamoxifen. AI are associated with hypercholesterolemia and increased risk of CVD mainly in patients at high baseline risk [64]. The mechanism that may explain the increase in CVD with the use of AI might involve the reduction of circulating estrogen levels and the consequent decrease in their positive effect on lipid metabolism, atherosclerosis and vascular tone [64]. In an Arimidex, Tamoxifen, Alone or in Combination (ATAC) trial, the incidence of CV events in women with previous heart disease was 17% in the AI group compared to 10% in the tamoxifen group [65]. These data were confirmed in subsequent metanalysis, especially when AI were tested in a head-to-head comparison with tamoxifen, even more than when compared to placebo. These data suggest that the remarkable adverse CV effects of AI compared to tamoxifen can be partially justified by the cardioprotective effect of tamoxifen itself [66]. A recent population-based cohort study, which included 1,7992 patients (8139 taking an AI and 9873 taking tamoxifen), showed a trend towards a higher risk in myocardial infarction and stroke and an increased risk of heart failure and CV mortality in the AI treated group [67]. These findings were confirmed by Matthews et al., who analyzed data from the United Kingdom and the United States registry of postmenopausal women with a diagnosis of breast cancer. They found an increase in CV outcome in AI users compared to tamoxifen treated patients. In this analysis, it has been consistently suggested that the observed difference in CV events could be explained by the cardioprotective effects of tamoxifen rather than an adverse effect of AI on the CV system [59]. A few small randomized control trials suggested that the sequential therapeutic regimen carries an increased risk of CV safety outcomes compared to the upfront regimen, but these data have not been confirmed in larger trials [68,69]. 

## 5. Targeted Therapy

### 5.1. Immune Checkpoint Inhibitors (ICI)

Pembrolizumab, Ipilimumab, Nivolumab and Atezolizumab are recent drugs that act through the enhancement of the body’s immune response against cancer [70]. The immune checkpoint inhibitors tested in breast cancer are Atezolizumab and Pembrolizumab [71]. They have been used in advanced triple negative breast cancer due to their ability to block the PDL1 binding site on tumor cells. The interaction between PD1 on T cell receptors and PDL1 on tumor cells causes the inhibition of the activation of T cells, which become unable to remove tumor cells [72]. The blockade of PDL1 by ICIs leads to a break in the immune tolerance of T cells, allowing them to cause tumor cell apoptosis [73]. It has been shown that ICIs can cause adverse CV events, such as arrhythmias, coronary artery disease, vasculitis and pericarditis, but the most common type of cardiotoxicity is myocarditis, representing 45% of adverse CV effects [74]. It has been suggested that the unselective mechanism of the actions of ICIs can determine an excessive immune response, which also reduces tolerance to endogenous antigens. Myocarditis is frequently reversible but can vary from mild to fulminant forms. Some available data show a positive therapeutic response to high doses of corticosteroids [74]. 

### 5.2. HER2 Targeted Therapy

Almost 20% of breast cancer overexpresses epidermal growth factor receptor 2 (HER2), which is involved in cell growth and repair [75]. HER-2 positive breast cancer is aggressive and has high recurrence and death rates, mostly because of increased angiogenesis with a higher risk of metastasis [76]. Trastuzumab was the first drug approved in the treatment of HER2-positive breast cancer [77,78]. It is a humanized monoclonal antibody that binds to the extracellular portion of the HER2 inhibiting its signaling pathway, mainly through hampering the omo or eterodimerization of HER2 receptors, leading to a dysregulation of the mitogen-activated protein kinase (MAPK) and the phosphatidylinositol 3-kinase (PI3K)/protein kinase B (PI3K/Akt) pathway and subsequently inhibiting the cell cycle progression [79,80,81,82,83]. The receptor binding might favor the ubiquitination and then degradation of Erb2, downregulating the exposure on the cell’s surface membrane [84]. Furthermore, trastuzumab might activate an antibody-dependent cell cytotoxicity reaction that can lead to cancer cell death [85]. In early clinical trials trastuzumab showed the capacity, in comparison to standard chemotherapy, to prolong the disease progression time and to reduce the rate of death, therefore improving the survival rate. Indeed, the introduction of trastuzumab in early breast cancer therapy has reduced the risk of death by 33% and the risk of disease recurrence by 50% [86]. Despite this, early evidence found that a trastuzumab-based regimen might be associated with cardiac dysfunction with variable incidence from 4% [87] to 27% [78]. This early evidence has been subsequently confirmed by many studies and meta-analysis. The amount of LVEF reduction and the incidence of congestive heart failure varies in different studies. In a wide epidemiologic study involving 9535 women with early breast cancer of whom 2203 received trastuzumab, Chavez-McGregor et al. found that the incidence of heart failure was 29% compared to the 18.9% in non-trastuzumab users [88]. In 2011, Slamon et al. noticed a reduction of >10% of the LVEF in 19% of patients receiving trastuzumab in association with anthracyclines and in 11% of patients receiving trastuzumab and taxanes [89]. Patients taking trastuzumab, in their chemotherapeutic regimen, have a risk of developing symptomatic heart failure, varying roughly between 2 and 4% [89,90]. A recent case-control study by Yun et al. confirmed the increased risk of developing signs and symptoms of heart failure in those patients treated with trastuzumab in whom a LVEF <55% was found during the follow-up for therapy-induced cardiotoxicity [91]. In a large retrospective analysis published in 2021 by Battisti et al., the incidence of cardiotoxicity in patients treated with trastuzumab was 16.6%, but the development of symptomatic heart failure evaluated by NYHA classification was 5.0% [92]. The exact mechanism behind trastuzumab-induced cardiotoxicity is not completely understood. The main hypothesis is that the neuregulin (NRG)-ERBB pathway plays a fundamental role. NRG is a ligand of ERBB receptors and its signaling axis plays a key role in the growth, survival, proliferation and response to cardiomyocytes stress [93,94]. Indeed, a series of experimental data in ERBB2-deleted mice showed the development of dilatated cardiomyopathy or severe systolic dysfunction after pressure overload. This pivotal role of the NRG-ERBB pathway in response to stress damage can also explain why there seems to be an additive risk of myocardial damage in patients assuming both anthracyclines and trastuzumab. Indeed, the NRG-ERBB pathway is implicated in the response in redox damage caused by anthracycline administration [93,93]. Portera et al. demonstrated that 45% of patients, assuming a combined regimen of trastuzumab–anthracycline, experienced a reduction in LVEF greater than 15% compared to the baseline or a reduction in the EF below 50%, but only 9% experienced signs and symptoms of congestive heart failure [95]. Other risk factors in the development of trastuzumab cardiotoxicity include older age, hypertension, diabetes mellitus, reduction in estimated glomerular filtration rate, previous cardiac disease and baseline left ventricular systolic dysfunction [96]. Biomarkers (Troponin I and NT-pro-brain natriuretic peptide) [97] and echocardiographic parameters have been studied to predict the development of cardiac toxicity. To monitor the risk of trastuzumab-induced cardiotoxicity, it is recommended to perform a baseline CV risk factor assessment (such as hypertension, hyperlipidemia, tobacco use, presence of heart failure symptoms) [94] and a baseline echocardiography exam [98]. Subsequently it is fundamental to begin a surveillance program at least every 3 months during therapy and then 3–12 months after the completion of the treatment. This standard protocol could be obviously personalized based on the different findings and patient status. At each examination, echocardiography plays a primary role in the determination of the possible trastuzumab cardiotoxicity. Cardiotoxicity is defined as an absolute reduction of >10% (6% with 3D echocardiography) in the LVEF, to a value <50%; probable subclinical cardiotoxicity is defined as a decline of >10% (6% with 3D echocardiography) in LVEF, but with a global EF that remain ≥50%, with a lowering in global longitudinal strain (GLS) < 15%; possible subclinical cardiotoxicity is defined as the reduction of LVEF < 10% to a value < 50% or a decline in GLS > 15% compared to baseline [96]. According to the definition of cardiotoxicity, societies of cardiovascular imaging, such as European Association of Cardiovascular Imaging (EACVI) [99] and the British Society of Echocardiography (BSE) [96], have established that to have a more reliable and early diagnosis of myocardial damage in patients taking trastuzumab, it is necessary to use the most advanced techniques available in echocardiography, such as the estimation of tissue Doppler 3D assessment of LVEF and speckle tracking with the assessment of GLS as well as radial and circumferential strain if viable. Indeed, to detect early myocardial damage, these techniques appear to be more sensitive and less operator dependent than 2D LVEF [19,20].

ACEi and beta blockers have been studied for the management and prevention of LV dysfunction in small trials with promising results [13]. The MANTICOR Trial investigated the efficacy of bisoprolol and perindopril compared to placebo in the prevention of trastuzumab toxicity in a randomized trial with a 1:1:1 design. There was no difference in the primary outcome defined as the change in diastolic indexed ventricular volume due to adverse remodeling. Conversely, a positive effect, demonstrated by an attenuation in the reduction of LVEF, was noticed for bisoprolol, compared both to perindopril and placebo (*p*-value = 0.001) [100]. Another relevant finding was the higher rate of trastuzumab interruption in the placebo group compared both to bisoprolol and perindopril group (*p* = 0.03) [100]. A recent randomized trial by Guglin et al. demonstrated that, in a population of women with HER2-positive breast cancer treated with trastuzumab, both lisinopril and carvedilol could prevent trastuzumab cardiotoxicity and patients treated with these drugs experienced fewer trastuzumab interruptions [101]. Moreover, the Safe-Heart trial showed, in a population of patients with reduced LVEF, that HER2 targeted-therapies had a good cardiac safety profile, proven by a not significant change in LVEF at the end of the treatment. It is important to note that the patients in the Safe-Heart study were treated with beta-blockers and/or ACEi or ARB prior to the start of on-study HER2 target therapy [102]. However, it is essential to highlight that trastuzumab-related cardiotoxicity is largely reversible, not dose-dependent and unlikely to cause late sequential dysfunction [103].

Another drug used in HER2 positive cancer is trastuzumab emtansine, which is an antibody-conjugated drug with cytotoxic properties. Emtansine binds to tubulin, leading to the disruption of microtubule formation interrupting the cell cycle with consequent apoptosis and cell death. This drug has been studied in three main phase three trials, showing a lower level of cardiotoxicity compared to other chemotherapy regimens in breast cancer. Therefore, trastuzumab emtansine is now indicated in patients with prior treatment with trastuzumab and taxanes [104,105,106].

Pertuzumab is a humanized antibody that binds HER2 receptor on a different domain compared to trastuzumab and could therefore work in a synergistical manner. In 2015, Swain et al. compared two different regimen therapies, with and without pertuzumab. This study has shown a lower incidence of left ventricular dysfunction in the pertuzumab group compared to the group receiving trastuzumab, docetaxel and placebo (6.6% vs. 8.6%) [107]. Recently the Adjuvant Pertuzumab and Trastuzumab in Early HER2-Positive Breast Cancer Trial (APHINITY TRIAL) aimed to explore the effect of trastuzumab in association with pertuzumab, showing that the pertuzumab group had a lower percentage of heart failure compared to the trastuzumab/placebo group [108].

Lapatinib is a tyrosine kinase inhibitor that is capable of blocking the intracellular signaling pathway of HER2 receptor. In the Adjuvant Lapatinib and Trastuzumab for Early Human Epidermal Growth Factor Receptor 2-Positive Breast Cancer: Results from the Randomized Phase III Adjuvant Lapatinib and/or Trastuzumab Treatment Optimization Trial (ATTLO trial) the head-to-head comparison between lapatinib and trastuzumab shows lower cardiac adverse events in the lapatinib group in a one-year follow-up. Patients treated with lapatinib demonstrated more adverse noncardiac events such hepatic toxicity, diarrhea and cutaneous rush [109].

Two more recent tyrosine kinase inhibitors, afatinib and neratinib, seem to be well tolerated, but more investigation in breast cancer is needed [110].

### 5.3. Cyclin Dependent Kinase 4/6 (CDK 4/6) Inhibitors

Palbociclib, Ribociclib and abemaciclib have recently been developed to overcome cancer resistance to conventional chemotherapy. They are inhibitors of CDK 4/6, a class of serine/threonine kinase is of primary importance in the progression of cell cycle [111]. These kinases cooperate with Cyclin D1 and Cyclin D3 in the regulation of the transition from the G1 to the S phase in cell cycle progression. The transition between those two phases is controlled by the retinoblastoma protein (Rb), which is a key regulator of the E2F transcription factor. The role of the Cyclin D-CDK 4/6 complex is the phosphorylation of the Rb to enable the release of the E2F transcription factor and its translocation in the cell nucleus to drive the expression of several genes fundamental to the transition G1/S. In breast cancer, it has been demonstrated that both estrogen receptor, through the enhancement of transcription of Cyclin D1, and HER2, through the activation of the PI3K/Akt/mTOR pathway, could increase the rate of tumor progression. Loss of INK4 and Cip/Kip family (inhibitors of CDK4/6) and overexpression of CDK4/6 have also been noted in breast cancer. This intricate pathway seems to confer resistance to the commonly used therapies [112,113]. The only approved use of this class of drugs is the treatment of advanced hormone receptor positive breast cancer in combination with endocrine therapy. In several trials, those drugs have demonstrated positive outcomes in terms of survival [114,115,116]. The most common adverse effect of this drug is bone marrow suppression and, consequently, pancytopenia [117,118]. The CV concerns for the toxicity of this class of drugs is principally due to the possible effect of ribociclib prolonging the QT interval. In two trials [119,120] the prolongation of QT interval affected almost 9% of patients. These data lead to the current indication that suggests the administration of ribociclib only in patients with a QT baseline interval <450 msec. It is also important to avoid the association of ribociclib with other drugs potentially implicated in the prolongation of the QT interval. This association with QT prolongation has not been confirmed for the other CDK4/6 inhibitors that were only associated with rare and mild adverse cardiac events, such as atrial fibrillation or pericardial effusion. Another important aspect to highlight is the hepatic metabolism through the cytochrome (mainly CYP3A4) of these three drugs that can cause drug–drug interaction, even with antihypertensive and antiarrhythmic drugs or oral anticoagulants that are commonly used in CV clinical practice [121].

## 6. Radiotherapy

Radiation therapy is used in the treatment of many types of solid cancer. Most of the deaths not related to breast cancer may be attributed to CV mortality secondary to irradiation on great vessels, surrounding tissues and the heart itself. The absolute risk for the development of CV side effects during radiotherapy can only poorly be estimated due to the coadministration of cardiotoxic chemotherapy as a confounding factor [122]. The cardiotoxic effects of radiation therapy on CV system involves direct ionization and cell damage by radiation and by water radiolysis products [123]. Radiation can cause a variety of cardiac alterations, including premature coronary artery diseases, pericarditis and pericardial effusion, cardiomyopathy, valvular disease and arrhythmias. The risk of radiation-induced heart disease (RIHD) seems to be strictly dependent on the surface of heart exposed to radiation and amount received. However, modern radiation technique, dose diminishing and the reduction of irradiated heart volume in many solid tumors has substantially reduced the frequency of RIHD. Side effects may occur within a few days of radiation treatment, but most RIHD seems to appear many years after treatment. The incidence of RIHD is higher in patients given high doses of radiation or radiation therapy concurrent with doxorubicin. Radiation might also increase the development of reactive oxygen species that cause proinflammatory states, leading to impaired healing and endothelial dysfunction. Endothelial dysfunction may lead to intimal thickening and accelerated atherosclerosis, especially in the coronary ostia [124].

Finally, it must be said that patients with active cancer are at an increased risk of arterial and venous thromboembolism and bleeding events. Indeed, by several prothrombotic mechanisms, including platelet activation, the increased expression of procoagulants and the suppression of fibrinolytic activity, malignancies might deeply affect the Virchow triad [125]. In the past, the risk of deep vein thrombosis and pulmonary embolism were extensively studied. Recently, it was also shown that the presence of a growing cancer, independent of treatments, might be associated with increased incidence of nonobstructive coronary arteries (MINOCA). Indeed, a recent report showed that the prevalence of malignancy in patients with MINOCA is not trivial and is significantly greater than in patients affected by myocardial infarction with coronary obstruction [126,127].

## 7. Conclusions

CV toxicity is a worrying side effect of most chemotherapeutic agents used to treat breast cancer. These agents can mainly cause LV systolic dysfunction leading to heart failure but can also expose patients to arrhythmic, ischemic and thromboembolic risks through molecular pathways, which need further investigation to be clearly elucidated. Hence, it is fundamental to assess CV risk before starting chemotherapies and to provide a strict follow-up of patients in order to enable the early detection of the signs and symptoms of cardiotoxicity due to chemotherapy. In this regard, the development of an integrated cardio-oncological flow chart is of fundamental importance. Moreover, the better understanding of the mechanisms of the actions of chemotherapies and their possible interference with the CV system is crucial in order to minimize their potentially deleterious impact on CV health.

## Figures and Tables

**Table 1:** Definitions of cardiotoxicity, modality of evaluation and chemotherapy agents.

	Definition	Modality of Evaluation	Chemotherapy Agents
Alexander et al. [12]	Mild: decline in LVEF > 10%. Moderate: decline in LVEF > 15% to final LVEF < 45% Severe: symptoms of congestive HF	MUGA scan	Anthracycline
Schwartz et al. [17]	Decline in LVEF > 10% to final LVEF < 50%	MUGA scan	Anthracycline
Seidman et al. [18]	1. Cardiomyopathy characterized by a decrease in LVEF globally or more severe in the septum 2. Sign and symptoms of HF 3. Decline of LVEF ≥ 5% to final EF < 55% with symptoms of congestive HF 4. Asymptomatic decline of LVEF ≥ 10% to final EF < 55%	Echocardiogram and MUGA scan	Trastuzumab +/− Anthracycline
Zamorano et al. [13]	1. Decline in LVEF >10% to a value < 50% 2. GLS > 15% relative percentage reduction	Two-dimensional (2D) and three-dimensional (3D) contrast echocardiography, cardiac magnetic resonance imaging, MUGA scan	N/A
Curigliano, G et al. [19]	Reduction in LVEF of 10%, especially if the number is below LVEF < 50%	Two-dimensional (2D) and three-dimensional (3D) contrast echocardiography, cardiac magnetic resonance imaging, MUGA scan	N/A

EF, ejection fraction; GLS, global longitudinal strain; HF, heart failure; LVEF, left ventricle ejection fraction; MUGA, multigated acquisition.

**Table 2:** Risk factors for anthracycline induced cardiotoxicity [20].

Risk Factors	Risk Level
Congestive heart failure	Very High
Ischemic cardiomyopathy	High
LVEF reduction	High
Elevated baseline troponin	High
Previous anthracycline treatment	High
Prior radiotherapy to left chest or mediastinum	High
Elevated baseline BNP or NT-proBNP	High
Age ≥ 80 years	High
Age 65–79 years	Medium
Baseline LVEF 50–54%	Medium
Hypertension	Medium
Diabetes	Medium
Chronic kidney disease	Medium
Previous nonanthracycline-based chemotherapy	Medium
Current smoker or smoking history	Medium
Obesity	Medium

BNP, brain natriuretic peptide; NT-proBNP, N-terminal prohormone of brain natriuretic peptide; LVEF, left ventricle ejection fraction; BMI, body mass index.

**Table 3:** Risk factors for anti-HER2-induced cardiotoxicity ([13,20]).

Anti-HER2 Agents/Tyrosine Kinase Inhibitor	Risk Factors
Trastuzumab Pertuzumab T-DMI Lapatinib	Age (>65 years) BMI > 30 kg/m^2^ Arterial Hypertension Left Ventricular Dysfunction (LVEF < 50%) Previous or roncomitant anthracycline treatment Previous Radiation Therapy (Left chest or Mediastinum) Heart Failure or Cardiomyopathy History of CAD (previous MI, CABG or coronary revascularization) Valvular Heart Disease Arrhythmia Elevated Cardiac Biomarkers (NT-pro-BNP, BNP, Troponin)

BMI, body mass index; BNP, brain natriuretic peptide; CABG, Coronary Artery Bypass Graft; CAD, Coronary Artery Disease; LVEF, left ventricle ejection fraction; MI, Myocardial Infarction; NT-proBNP, N-terminal prohormone of brain natriuretic peptide.

**Table 4:** Different dosages of anthracyclines and incidence of left ventricular dysfunction [13].

Anthracycline	Incidence of LV Dysfunction (%)
Doxorubicin 400 mg/m^2^	3–5
Doxorubicin 550 mg/m^2^	7–26
Doxorubicin 700 mg/m^2^	18–48
Epirubicin > 900 mg/m^2^	0.9–11.4
Liposomal anthracyclines > 900 mg/m^2^	2

LV, left ventricular.

## Data Availability

Not applicable.
